# Evolution and expansion of multidrug-resistant malaria in southeast Asia: a genomic epidemiology study

**DOI:** 10.1016/S1473-3099(19)30392-5

**Published:** 2019-09

**Authors:** William L Hamilton, Roberto Amato, Rob W van der Pluijm, Christopher G Jacob, Huynh Hong Quang, Nguyen Thanh Thuy-Nhien, Tran Tinh Hien, Bouasy Hongvanthong, Keobouphaphone Chindavongsa, Mayfong Mayxay, Rekol Huy, Rithea Leang, Cheah Huch, Lek Dysoley, Chanaki Amaratunga, Seila Suon, Rick M Fairhurst, Rupam Tripura, Thomas J Peto, Yok Sovann, Podjanee Jittamala, Borimas Hanboonkunupakarn, Sasithon Pukrittayakamee, Nguyen Hoang Chau, Mallika Imwong, Mehul Dhorda, Ranitha Vongpromek, Xin Hui S Chan, Richard J Maude, Richard D Pearson, T Nguyen, Kirk Rockett, Eleanor Drury, Sónia Gonçalves, Nicholas J White, Nicholas P Day, Dominic P Kwiatkowski, Arjen M Dondorp, Olivo Miotto

**Affiliations:** a Wellcome Sanger Institute, Hinxton, UK; b Cambridge University Hospitals NHS Foundation Trust, Cambridge, UK; c MRC Centre for Genomics and Global Health, Big Data Institute, University of Oxford, Oxford, UK; d Mahidol-Oxford Tropical Medicine Research Unit, Mahidol University, Bangkok, Thailand; e Centre for Tropical Medicine and Global Health, Nuffield Department of Medicine, University of Oxford, Oxford, UK; f Institute of Malariology, Parasitology and Entomology, Quy Nhon, Vietnam; g Oxford University Clinical Research Unit, Ho Chi Minh City, Vietnam; h Centre of Malariology, Parasitology, and Entomology, Vientiane, Laos; i Institute of Research and Education Development, University of Health Sciences, Vientiane, Laos; j Lao-Oxford-Mahosot Hospital Wellcome Trust Research Unit (LOMWRU), Vientiane, Laos; k National Center for Parasitology, Entomology, and Malaria Control, Phnom Penh, Cambodia; l Laboratory of Malaria and Vector Research, National Institute of Allergy and Infectious Diseases, National Institutes of Health, Rockville, MD, USA; m Provincial Health Department, Pailin, Cambodia; n Faculty of Tropical Medicine, Mahidol University, Bangkok, Thailand; o The Royal Society of Thailand, Bangkok, Thailand; p Worldwide Antimalarial Resistance Network (WWARN), Asia Regional Centre, Bangkok, Thailand; q Harvard TH Chan School of Public Health, Harvard University, Boston, MA, USA; r Wellcome Centre for Human Genetics, University of Oxford, Oxford, UK

## Abstract

**Background:**

A multidrug-resistant co-lineage of *Plasmodium falciparum* malaria, named KEL1/PLA1, spread across Cambodia in 2008–13, causing high rates of treatment failure with the frontline combination therapy dihydroartemisinin-piperaquine. Here, we report on the evolution and spread of KEL1/PLA1 in subsequent years.

**Methods:**

For this genomic epidemiology study, we analysed whole genome sequencing data from *P falciparum* clinical samples collected from patients with malaria between 2007 and 2018 from Cambodia, Laos, northeastern Thailand, and Vietnam, through the MalariaGEN *P falciparum* Community Project. Previously unpublished samples were provided by two large-scale multisite projects: the Tracking Artemisinin Resistance Collaboration II (TRAC2) and the Genetic Reconnaissance in the Greater Mekong Subregion (GenRe-Mekong) project. By investigating genome-wide relatedness between parasites, we inferred patterns of shared ancestry in the KEL1/PLA1 population.

**Findings:**

We analysed 1673 whole genome sequences that passed quality filters, and determined KEL1/PLA1 status in 1615. Before 2009, KEL1/PLA1 was only found in western Cambodia; by 2016–17 its prevalence had risen to higher than 50% in all of the surveyed countries except for Laos. In northeastern Thailand and Vietnam, KEL1/PLA1 exceeded 80% of the most recent *P falciparum* parasites. KEL1/PLA1 parasites maintained high genetic relatedness and low diversity, reflecting a recent common origin. Several subgroups of highly related parasites have recently emerged within this co-lineage, with diverse geographical distributions. The three largest of these subgroups (n=84, n=79, and n=47) mostly emerged since 2016 and were all present in Cambodia, Laos, and Vietnam. These expanding subgroups carried new mutations in the *crt* gene, which arose on a specific genetic background comprising multiple genomic regions. Four newly emerging *crt* mutations were rare in the early period and became more prevalent by 2016–17 (Thr93Ser, rising to 19·8%; His97Tyr to 11·2%; Phe145Ile to 5·5%; and Ile218Phe to 11·1%).

**Interpretation:**

After emerging and circulating for several years within Cambodia, the *P falciparum* KEL1/PLA1 co-lineage diversified into multiple subgroups and acquired new genetic features, including novel *crt* mutations. These subgroups have rapidly spread into neighbouring countries, suggesting enhanced fitness. These findings highlight the urgent need for elimination of this increasingly drug-resistant parasite co-lineage, and the importance of genetic surveillance in accelerating malaria elimination efforts.

**Funding:**

Wellcome Trust, Bill & Melinda Gates Foundation, UK Medical Research Council, and UK Department for International Development.

## Introduction

In recent years, frontline treatments for *Plasmodium falciparum* malaria have been failing in parts of southeast Asia,^1^, ^2^, ^3^ a historic epicentre for the emergence and spread of antimalarial drug resistance.^4^ The current treatment for *P falciparum* consists of a fast-acting artemisinin derivative and a longer-acting partner drug, termed artemisinin combination therapy. Dihydroartemisinin with piperaquine has been the artemisinin combination therapy of choice in Cambodia, Thailand, and Vietnam for lengthy periods during the past decade. By 2008, parasites in western Cambodia began developing resistance to dihydroartemisinin-piperaquine, manifesting first through delayed clearance in response to artemisinins^5^, ^6^, ^7^, ^8^, ^9^ (which might have begun several years earlier), and later with the addition of resistance to the partner drug piperaquine.^1^, ^3^, ^8^ By 2013, dihydroartemisinin-piperaquine was failing to clear *P falciparum* infections in 46% of patients treated in western Cambodia.^3^ This resistance arose on a background of pre-existing resistance to multiple antimalarial drugs, leaving few treatment options and threatening plans for malaria elimination in the region.

Large-scale genetic analyses have revealed the detailed epidemiology of drug resistance,^10^, ^11^, ^12^, ^13^, ^14^, ^15^ complementing the clinical observation of increasing rates of treatment failure. Non-synonymous mutations in *kelch13*—the most prevalent of which is the Cys580Tyr (C580Y) mutation^9^, ^11^, ^16^—have proved to be valuable markers for tracking artemisinin resistance, as has amplification of *plasmepsin 2*/*3*^17^, ^18^, ^19^, ^20^ in tracking piperaquine resistance. The frequency of these genetic markers increased across the eastern Greater Mekong Subregion from 2008 to 2015,^12^, ^13^, ^14^, ^15^ corresponding with the spread of dihydroartemisinin-piperaquine treatment failure. Whole genome sequencing has provided deeper insight into the movement, demographics, and evolution of resistant parasites.^12^, ^13^ Detailed analyses of a large whole genome dataset, including samples up to 2013, revealed that most parasites with the *kelch13* C580Y mutation and amplification of *plasmepsin 2/3* were derived from a single parasite co-lineage, termed KEL1/PLA1, that arose in western Cambodia.^13^

**Research in context**

**Evidence before this study**

This study updates our previous work describing the emergence and spread of a multidrug-resistant *Plasmodium falciparum* co-lineage (KEL1/PLA1) within Cambodia up to 2013. A regional genetic surveillance project, Greater Mekong Subregion (GenRe-Mekong), has since reported increased frequency of dihydroartemisinin-piperaquine resistance markers in neighbouring countries. We searched PubMed using the terms “artemisinin”, “piperaquine”, “resistance”, and “southeast Asia” for articles published since our previous study, from Oct 30, 2017, to Jan 5, 2019. Our search yielded 28 results, including reports of a recent sharp decline in the clinical efficacy of dihydroartemisinin-piperaquine in Vietnam; the spread of genetic markers of dihydroartemisinin-piperaquine resistance into neighbouring countries; and reports associating mutations in the *crt* gene with piperaquine resistance, including newly emerging *crt* variants in southeast Asia.

**Added value of this study**

In this genomic epidemiology study, we analysed *P falciparum* whole genomes collected up to early 2018 from eastern southeast Asia (the geographical region comprising Cambodia, southern Laos, northeastern Thailand, and southern and central Vietnam). We describe the fine-scale epidemiology of KEL1/PLA1 genetic subgroups that have spread from Cambodia since 2015 and taken over indigenous parasite populations across eastern southeast Asia. Several newly emerging *crt* mutations accompanied the spread and expansion of KEL1/PLA1 subgroups, suggesting a proliferation of biologically fit, multidrug-resistant parasites.

**Implications of all the available evidence**

The problem of *P falciparum* multidrug resistance has substantially worsened in eastern southeast Asia since previous reports. KEL1/PLA1 has diversified and spread widely across the region since 2015, becoming the predominant parasite group in several of the endemic areas surveyed. This expansion might have been fuelled by continued exposure to dihydroartemisinin-piperaquine, resulting in sustained selection after KEL1/PLA1 became established. Continued drug pressure enabled the acquisition of further mutations, resulting in higher levels of resistance. These data show the value of genetic surveillance of pathogens and the urgent need to eliminate these dangerous parasites.

Such analyses raised uncertainties surrounding the future of KEL1/PLA1. Would these parasites continue their aggressive spread out from Cambodia? Would they spread clonally or heterogeneously? Could they evolve even higher levels of resistance or improved fitness? Newly emerging mutations in the *crt* gene have been reported to cause piperaquine resistance in vitro.^21^, ^22^, ^23^ These *crt* substitutions occurred on a *plasmepsin 2*/*3* amplified background, raising the question of how mutations at multiple loci interact to produce resistant phenotypes; where and by what process the new *crt* mutations are spreading; and how these mutations relate to the evolution and expansion of KEL1/PLA1. To address these questions, we investigated the genomic epidemiology of parasites resistant to dihydroartemisinin-piperaquine using the most recent *P falciparum* genomic dataset currently available, including samples up to early 2018, collected across the region through the MalariaGEN *P falciparum* Community Project.

## Methods

### Study design

In this genomic epidemiology study we analysed whole genome sequence data from samples in the MalariaGEN *P falciparum* Community Project Pf6.2 data release. A large proportion of samples were collected in clinical studies, as detailed in previous publications.^1^, ^9^, ^12^, ^13^ Previously unpublished samples were provided by two large-scale multisite projects: the Tracking Artemisinin Resistance Collaboration II (TRAC2) and the Genetic Reconnaissance in the Greater Mekong Subregion project (GenRe-Mekong, SpotMalaria). TRAC2 did drug efficacy trials at seven sites in eastern southeast Asia, contributing DNA from leukocyte-depleted venous blood samples taken from up to 120 symptomatic patients per site.^24^ GenRe-Mekong contributed dried blood spot samples from symptomatic patients with a positive rapid diagnostic test, collected by surveillance projects at public health facilities in multiple provinces of Cambodia, Laos, and Vietnam (appendix p 1). All patients provided informed consent through study protocols approved by the relevant local ethics authorities.

Ethical approval was obtained from the National Ethics Committee for Health Research, Ministry of Health, Phnom Penh, Cambodia; the Ministry of Health National Ethics Committee For Health Research, Laos; the Ethics Committee, Faculty of Tropical Medicine, Mahidol University, Bangkok, Thailand; the Ethical Committee, Hospital for Tropical Diseases, Ho Chi Minh City, Vietnam; and the Oxford Tropical Research Ethics Committee, Oxford, UK. No clinical or personal patient data were used in this analysis.

### Data collection and analysis

DNA from dried blood spot samples underwent selective whole genome amplification^25^ before sequencing. Sequence data were generated at the Wellcome Sanger Institute with Illumina short-read technology, and read counts at 1 043 334 quality-filtered biallelic single-nucleotide polymorphisms (SNPs) in the nuclear genome variants were called with a standardised analysis pipeline^26^ (Pf6 release). Genotypes were called only with a coverage of five or more reads and alleles were disregarded when represented by fewer than two reads, or 5% of reads when coverage was higher than 50.

To minimise errors and biases, we excluded from the analysis known or suspected duplicate samples, samples from time sequences and recurrences, samples sequenced with reads of fewer than 75 nucleotides, and those with insufficient coverage at more than 25% of the SNPs. After removing all SNPs that were invariant or had insufficient coverage in more than 25% of the remaining samples, we used 56 026 SNPs in our analysis. After estimating *F*_WS_ as previously described,^26^ we removed samples with *F*_WS_ less than 0·95, yielding 1673 essentially monoclonal samples for analysis. Of these, 466 (28%, largely from 2016–18) were obtained from dried blood spot specimens after selective whole genome amplification, whereas the remainder were genotyped without amplification.

### KEL1, PLA1, and *crt* haplotype classification

To identify *kelch13* mutations associated with artemisinin resistance, we scanned sequencing reads that align to *kelch13* amino acid positions 350 and above, identifying all non-synonymous variants. Samples without non-synonymous mutations were labelled as wild type, unless more than 25% of positions had insufficient coverage, in which case the sample was labelled as undetermined. Remaining samples were labelled according to the *kelch13* mutation found, or heterozygous if mutation sites were heterozygous. When identifying C580Y mutants, we disregarded samples that were heterozygous at that position. To assign membership to the KEL1 lineage, we tested its five characteristic SNPs.^13^ Moving away from *kelch13* and ignoring missing genotypes, we counted positions carrying KEL1 characteristic alleles, until a mismatch was encountered. Samples with three or more characteristic alleles were labelled as KEL1. PLA1 parasites were identified by scanning sequencing reads for the characteristic duplication breakpoint.^13^

A sample was assigned to one of the four newly emerging allele haplotypes if the corresponding position in *crt* was mutated, whereas the remaining three positions carried wild-type alleles. Remaining samples were assigned to the “no *crt*” group if all newly emerging allele positions were found to be wild type, and categorised as missing if the genotype could not be called at all the mutations.

### Population genomics analysis

Analyses were done with a combination of custom software programs written in Java, R, and Python with the toolkit Scikit-Allel. To study population structure in *N* samples, we constructed an N × N pairwise distance matrix using a previously published procedure.^12^ Analyses of relatedness were done with the module “cluster” from the python package “SciPy”, version 0.19.

### Statistical analysis

We applied the two-sided non-parametric Mann-Whitney *U* test with continuity correction to compare distributions of values, using p values less than 0·0001 as the Bonferroni-corrected significance threshold. p values less than 10^−16^ were not reported. All statistical analyses were done with the module ‘stats’ from the python package ‘SciPy’, version 0.19.

To control for spatial sampling heterogeneity, we used subsampling analyses of KEL1/PLA1 and *crt* frequency changes to balance region sample counts. We selected the earliest and latest pairs of consecutive years in which each region was represented by more than 50 samples (2010–11 and 2016–17), and estimated frequencies from 50 randomly selected samples per region. Northern Cambodia and northeastern Thailand were excluded because of insufficient samples. Median and IQR allele frequencies for the two time periods were calculated from 100 iterations.

### Role of the funding source

The funders had no role in study design, data collection, data analysis, data interpretation, or writing of the report. The corresponding author had full access to all the data in the study and had final responsibility for the decision to submit for publication.

## Results

We analysed a dataset of 2465 whole parasite genomes from the MalariaGEN *P falciparum* Community Project, collected in 2007–18 from Cambodia, Laos, northeastern Thailand, and Vietnam. This geographical region is referred to as eastern southeast Asia, the only region where KEL1/PLA1 has been found to date. After removing replicates, samples with low coverage, and highly diverse infections (*F*_WS_<0·95), we analysed a dataset of 1673 samples (appendix pp 1–3), in 1615 of which the KEL1/PLA1 status could be reliably identified.

**Figure 1:**
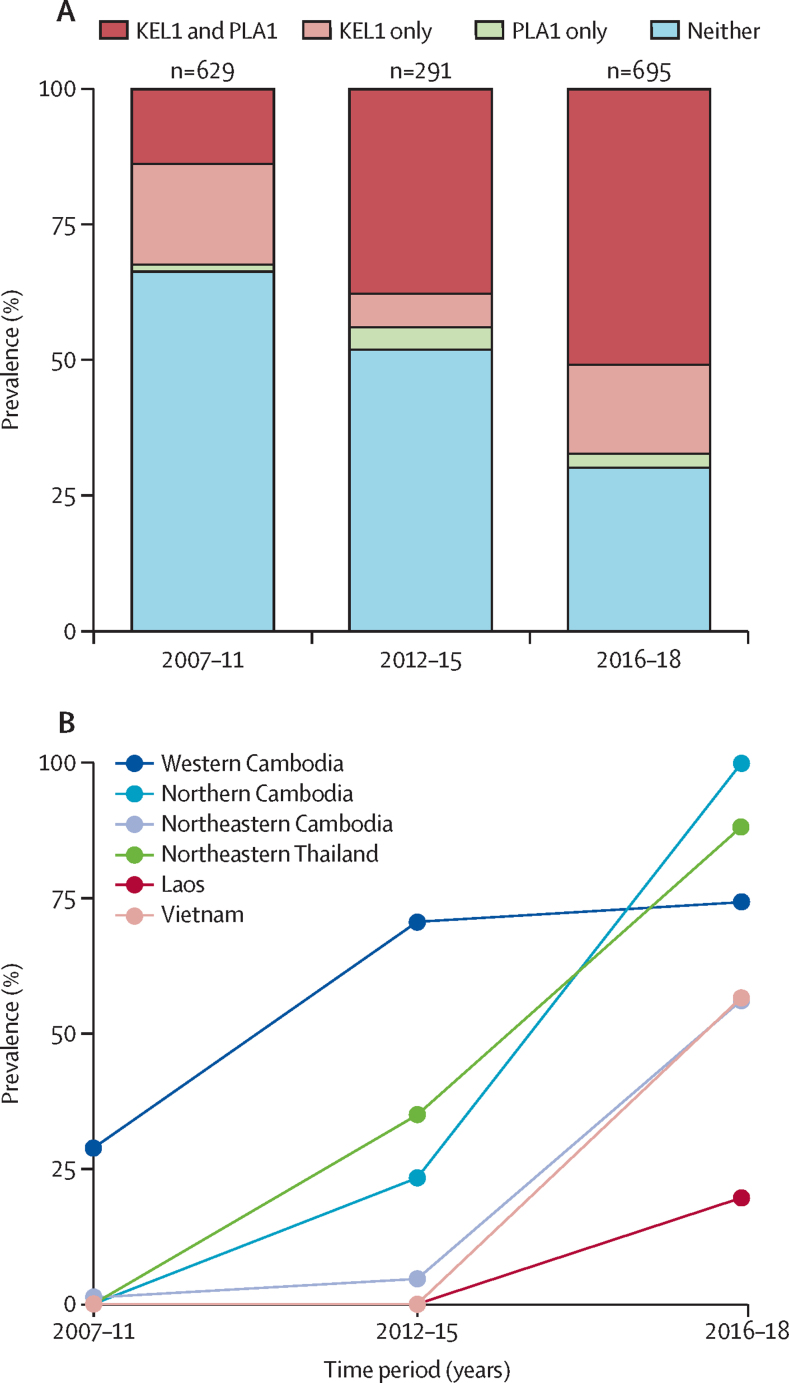
Rise in KEL1/PLA1 prevalence over time in eastern southeast Asia (A) Proportions of different combinations of KEL1 and PLA1 alleles, across three time periods (2007–11, 2012–15, and 2016–18) in the eastern southeast Asia regions surveyed in this study. (B) Change in the frequency of KEL1/PLA1 parasites during the same time periods in different geographical regions within eastern southeast Asia.

We identified 996 (60%) of 1673 samples as *kelch13* mutant parasites, of which 816 (82%) were C580Y. As previously reported,^11^ the *kelch13* mutations were mutually exclusive (no parasites harboured more than one mutation). The next most common *kelch13* mutants were Tyr493His (Y493H; 54 [5·4%] of 996) and Arg539Thr (R539T; 53 [5·3%] of 996). Most of C580Y mutants (802 [98%] of 816) belonged to the KEL1 lineage, denoting a specific haplotype surrounding the *kelch13* locus and a single epidemiological origin in western Cambodia.^13^ Of the KEL1 parasites, 551 (69%) of 802 mutants carried an amplification of the *plasmepsin 2/3* genes with a shared haplotype, here named PLA1, also consistent with a single epidemiological origin at this locus (appendix p 4). Overall the frequency of KEL1/PLA1 increased over the study period (figure 1). Co-occurrence of KEL1 with PLA1 increased significantly from 2007–11 (*r*^2^ 0·28) to 2016–18 (*r*^2^ 0·41), and more than half of parasites sampled in later years were KEL1/PLA1 (354 [51%] of 695), reflecting the expansion of this co-lineage (figure 1A). Before 2009, KEL1/PLA1 was only found in western Cambodia; by 2016–18 its prevalence had risen to higher than 50% in all regions sampled except for Laos (figure 1B; appendix p 5). This rapid rise was particularly notable in northeastern Thailand and Vietnam, where more than 80% of recent samples were KEL1/PLA1, despite their earlier absence from these areas, consistent with near-wholesale replacement of indigenous parasite populations. Increases in KEL1/PLA1 frequency in different regions and throughout eastern southeast Asia were confirmed after correcting for uneven sampling across regions (appendix pp 6–7).

**Figure 2:**
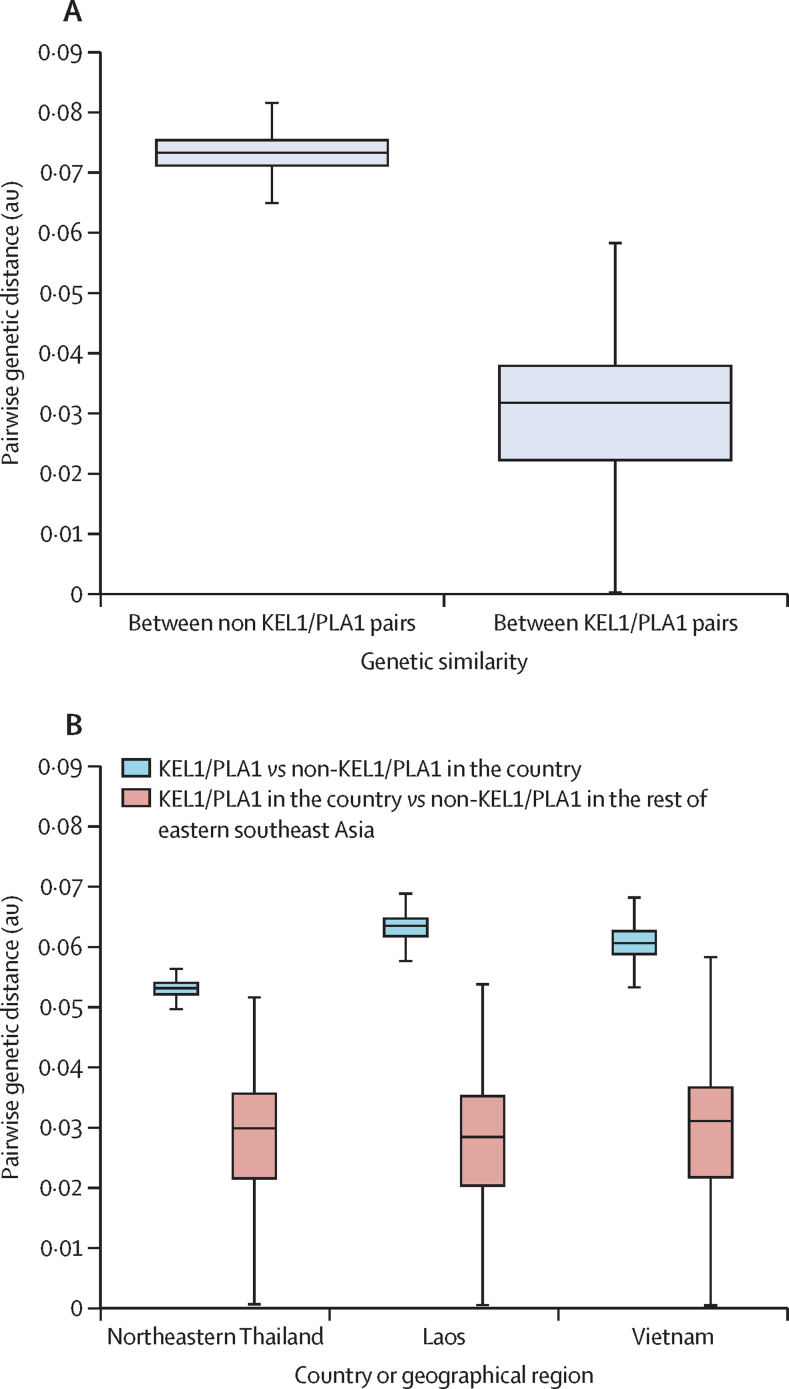
Genetic similarity among KEL1/PLA1 parasites across geographical regions (A) Boxplot comparing the distribution of pairwise genetic distance in non-KEL1/PLA1 parasites (ie, carrying neither KEL1 nor PLA1 haplotypes, n=777) with the distribution in KEL1/PLA1 parasites (n=551). (B) Boxplot comparing the distribution of pairwise distance between KEL1/PLA1 and non-KEL1/PLA1 parasites in the same geographical region (blue); and between KEL1/PLA1 parasites in the region and KEL1/PLA1 parasites outside the region (red). The number of samples analysed (in the following order: KEL1/PLA1 in the region, KEL1/PLA1 outside the region, and non-KEL1/PLA1 in the region) was 22, 529, and 14 in northeastern Thailand; 32, 519, and 193 in Laos; and 162, 389, and 207 in Vietnam. In both plots, pairwise genetic distance is expressed in an arbitrary unit, which is a function of the number of genetic differences observed among variant single-nucleotide polymorphisms (SNPs) in this dataset between pairs of samples, after correcting for linkage disequilibrium and heterozygous genotypes. Thick lines represent median values, boxes show the IQR, and whiskers represent extremes of the distribution, discounting outliers.

In previous work, we showed that KEL1/PLA1 parasites from northeastern Cambodia were genetically similar to those from western Cambodia, consistent with spread from western Cambodia.^13^ We extended this analysis by examining genetic similarity between parasites across the entire eastern southeast Asia region. Overall, KEL1/PLA1 parasites had lower genetic diversity than non-KEL1/PLA1 parasites (median 0·032 *vs* 0·073; p<10^−16^, Mann-Whitney *U* test; figure 2A; appendix p 8). Importantly, KEL1/PLA1 parasites were genetically more similar to each other than to non-KEL1/PLA1 parasites, regardless of their geographical origins; for example, KEL1/PLA1 parasites from Vietnam were more similar to KEL1/PLA1 parasites from other regions than to other types of parasites from Vietnam (p<10^−16^ for all comparisons, Mann-Whitney *U* test; figure 2B; appendix p 9). This observation is consistent with KEL1/PLA1 being an invading population with origins in western Cambodia and spreading into surrounding countries.

**Figure 3:**
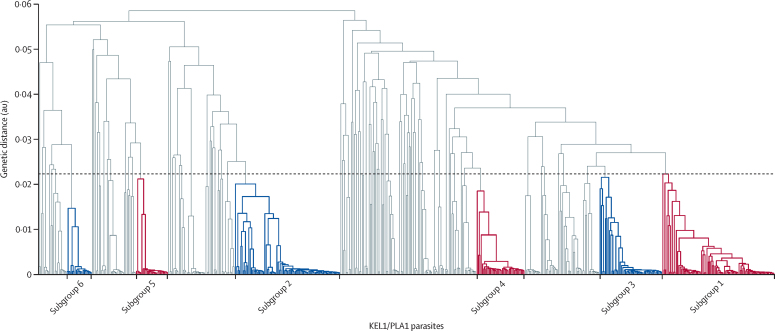
KEL1/PLA1 family tree The dendrogram shows a hierarchical clustering tree of pairwise genetic distances for all 551 KEL1/PLA1 samples across eastern southeast Asia; longer branches indicate more distant relationships. The six largest subgroups of highly related parasites are shown in red and blue, and labelled below the tree. The alternating colours highlight the different subgroups. These subgroups, numbered in order of decreasing size (subgroup 1, n=84; subgroup 2, n=79; subgroup 3, n=47; subgroup 4, n=36; subgroup 5, n=24; and subgroup 6, n=19), were identified by grouping samples with pairwise genetic distances in the lowest quartile (delimited by a dotted line). Pairwise genetic distance is expressed in an arbitrary unit, which is a function of the number of genetic differences observed among variant single-nucleotide polymorphisms (SNPs) in this dataset between pairs of samples, after correcting for linkage disequilibrium and heterozygous genotypes.

Given the high degree of genetic similarity between KEL1/PLA1 parasites, we investigated whether these parasites have spread through a single clonal expansion or as multiple independent subgroups. Hierarchical clustering of pairwise genetic distances was used to identify groups of closely related KEL1/PLA1 parasites (figure 3). We defined subgroups of related parasites whose pairwise genetic distance was in the lower quartile of the KEL1/PLA1 population (appendix p 10) and numbered these subgroups (ordered by size). The six largest subgroups together comprised more than 50% of KEL1/PLA1 samples, and broadly captured the largest expansions of near-identical parasites, with low genetic diversity within each subgroup (appendix p 11). The subgroups had distinct geographical, temporal, and genetic properties, reflecting separate epidemiological and evolutionary histories (appendix pp 12–13).

**Figure 4:**
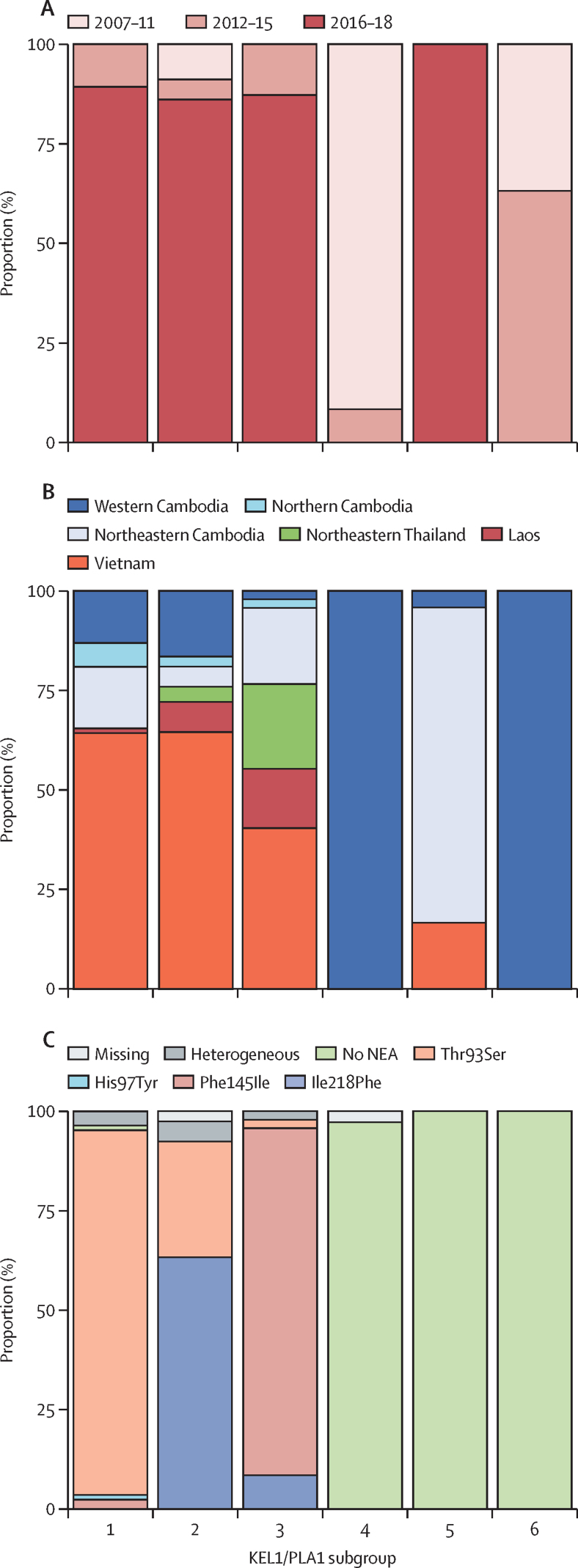
Distinct epidemiological and genetic properties of KEL1/PLA1 subgroups Sample proportions by sampling time period (A) and location (B) in the six largest groups of high-similarity KEL1/PLA1 parasites. Subgroups 1–3 emerged recently and are internationally distributed, whereas subgroups 4 and 6 are older and confined to western Cambodia. Proportion of *crt* haplotypes in the same groups (C): newly emerging *crt* mutations are highly prevalent in the newer subgroups 1–3, but absent from the older geographically restricted subgroups 4 and 6, and also in subgroup 5, which has recently expanded in northeastern Cambodia. Numbers of samples are as follows: n=84 for subgroup 1, n=79 for subgroup 2, n=47 for subgroup 3, n=36 for subgroup 4, n=24 for subgroup 5, and n=19 for subgroup 6. Together, these samples comprise more than 50% of the 551 analysed KEL1/PLA1 samples.

Subgroups 1 (n=84), 2 (n=79), and 3 (n=47) mostly emerged since 2016 and were all present in Cambodia, Laos, and Vietnam (figure 4A, 4B). These larger KEL1/PLA1 subgroups were not geographically restricted and co-existed simultaneously at the same locations across eastern southeast Asia. This combination of high genetic similarity and broad geographical dispersal over a few years implies rapid proliferation and expansion in independent overlapping transmission waves, suggesting that these parasites possess a selective advantage. By contrast, subgroup 4 (n=36) and subgroup 6 (n=19) were largely confined to Cambodia; they were responsible for the initial KEL1/PLA1 expansion in 2007–11, but subsequently became uncommon. We also identified smaller subgroups with limited geographical and temporal distributions, such as subgroup 5 (n=24), which was almost exclusively found in northeastern Cambodia in 2016–17.

The recent, rapid international expansion of some KEL1/PLA1 subgroups after years of confinement in Cambodia raises the question of whether new genetic changes have produced advantageous phenotypic effects in these subgroups. One candidate set of driver mutations are substitutions in the *crt* gene (PF3D7_0709000) that were recently associated with piperaquine resistance.^22^ To investigate this possibility, we compared allele frequencies for all non-synonymous *crt* SNPs in an earlier sampling interval (2010–11) and a later interval (2016–17), after correcting for uneven sampling across regions (appendix p 14). Four of these mutations (Thr93Ser [T93S], His97Tyr [H97Y], Phe145Ile [F145I], and Ile218Phe [I218F]), here referred to as newly emerging alleles, were rare in the early period (frequency ≤1%) and became more common (frequency ≥5%) by 2017–18 (T93S rising to 19·8%, H97Y to 11·2%, F145I to 5·5%, and I218F to 11·1%). These mutations were mutually exclusive: in the entire analysis dataset, we did not identify any sample carrying multiple newly emerging alleles, despite their simultaneous presence in the same geographical regions.

We found that newly emerging alleles occurred on a specific constellation of other, more prevalent *crt* mutations, comprising Lys76Thr (K76T) and other mutations in common with all chloroquine-resistant parasites^27^ (appendix p 15). Newly emerging alleles were only found on parasites possessing the most common chloroquine-resistant haplotype in eastern southeast Asia (CVIET, named by *crt* amino acid positions 72–76), plus mutations Asn326Ser and Ile356Thr, previously associated with artemisinin-resistant *kelch13* variants.^11^ Additionally, newly emerging alleles were mainly found in KEL1/PLA1 parasites (323 [78%] of 414 parasites with newly emerging alleles were also KEL1/PLA1). Consistent with the spread of KEL1/PLA1 and rising frequency of newly emerging alleles, SNPs associated with the CVIET haplotype all increased in frequency over the study period at the expense of those in other *crt* haplotypes (appendix p 14).

Three of the newly emerging alleles—T93S, F145I, and I218F—were embedded within long shared haplotypes, with reduced genetic diversity across the whole of chromosome 7 (appendix pp 16–18). This denotes limited breakdown through recombination, typical of a very recent selective sweep. Consistent with a recent emergence, these newly emerging alleles were mainly found in newer KEL1/PLA1 subgroups: T93S was near fixation in subgroup 1, as was F145I in subgroup 3, whereas subgroup 2 contained a mixture of T93S and I218F parasites (figure 4C; appendix pp 12–13). H97Y was distributed across multiple KEL1/PLA1 subgroups, had shorter haplotypes surrounding *crt*, and the parasites had higher levels of genetic diversity than the other newly emerging alleles, suggesting more extensive recombination. In summary, our data suggest that multiple KEL1/PLA1 subgroups were able to spread rapidly across borders in separate transmission waves, following the acquisition of one of several mutually exclusive *crt* mutations, which have emerged on a complex genetic background, including a constellation of other *crt* mutations that have accumulated over decades in eastern southeast Asia.

Northeastern Thailand provides a case study in the genomic epidemiology of these spreading multidrug-resistant parasites. In 2011, all parasites sampled from northeastern Thailand were *kelch13* R539T mutants, and possessed neither *plasmepsin 2/3* amplification nor any newly emerging alleles in *crt*. Although they had slow parasite clearance times,^9^, ^11^, ^16^ dihydroartemisinin-piperaquine remained an effective treatment because of sensitivity to piperaquine. These parasites had exceptionally low genetic diversity (appendix p 8), perhaps reflecting population collapse because of effective malaria control efforts. By 2017, however, KEL1/PLA1 had entirely replaced the R539T population, with a corresponding rise in dihydroartemisinin-piperaquine resistance. Although the majority of these parasites were from subgroup 3 and possessed the *crt* F145I mutation, we found other newly emerging alleles in this area, suggesting that multiple enhanced KEL1/PLA1 subgroups, possessing distinct *crt* mutations, invaded northeastern Thailand independently and replaced earlier parasite populations.

## Discussion

After a decade of progress,^28^ malaria incidence and mortality have been increasing since 2015, putting global malaria targets at risk.^29^ Major challenges include inadequate funding, parasite drug resistance, and insecticide resistance in mosquito vectors.^30^ Previous work has described a worrying situation unfolding in southeast Asia over the 2007–13 period, with the emergence of a dominant parasite co-lineage, KEL1/PLA1, that spread across Cambodia and caused dihydroartemisinin-piperaquine treatment failure.^13^ We describe the ongoing evolution and expansion of multidrug-resistant *P falciparum*, using whole genomes sampled across eastern southeast Asia and collected up to early 2018.

Our data clearly show that KEL1/PLA1 has continued spreading out from western Cambodia and is now highly prevalent in multiple regions of Laos, Thailand, and Vietnam, where it has frequently replaced previous indigenous populations of parasites. At all locations, KEL1/PLA1 parasites were genetically distinct from non-KEL1/PLA1 parasites, reflecting their recent shared ancestry. Genomic data show that underlying this spread is not a single lineage, but instead multiple subgroups of KEL1/PLA1 parasites, which have spread across eastern southeast Asia in independent transmission waves. These subgroups carry newly emerging alleles in the *crt* gene, which have arisen on a specific constellation of background *crt* mutations, most frequently in KEL1/PLA1 parasites.

The rapid rise in the frequency of these *crt* alleles suggests that they are markers of an advantageous phenotype. Two newly emerging alleles (F145I and H97Y) have been shown to reduce piperaquine sensitivity in vitro,^22^ and a new clinical study^24^ shows that H97Y, F145I, and I218F are associated with a higher rate of dihydroartemisinin-piperaquine treatment failures. Other *crt* alleles arising on a similar genetic background might also be functionally significant—for example, Gly353Val (G353V) has been associated with reduced piperaquine sensitivity in vitro.^22^ Thus, several novel *crt* variants might be capable of reducing parasite sensitivity to piperaquine, and among these the newly emerging alleles are those whose recent rise in frequency is most conspicuous in our dataset. Parasites harbouring piperaquine-resistant *crt* mutations, including F145I and G353V, were out-competed in vitro during asexual blood-stage development by lab isolates without the mutations, in the absence of drug pressure.^22^ The rise in frequency of these variations, in spite of fitness cost, is further evidence that they confer an increased survival advantage under strong and sustained piperaquine pressure.

Vietnam has used dihydroartemisinin-piperaquine as first-line treatment since 2004, Cambodia during the 2008–16 period, and Thailand since 2015. Cambodia has since adopted artesunate-mefloquine as first-line treatment, whereas the other two countries are reviewing current policy and procedures. Starting around 2008, KEL1/PLA1 parasites were first detected in western Cambodia and then expanded within Cambodia. They progressively replaced local parasite populations, such that by 2014 nearly all parasites sampled from western Cambodia were KEL1/PLA1.^13^ This replacement was probably driven by resistance to dihydroartemisinin-piperaquine, consistent with the association between *plasmepsin 2/3* amplification and piperaquine resistance in parasites collected before newly emerging alleles rose in frequency.^17^, ^18^ We propose that, after several years of continued exposure to dihydroartemisinin-piperaquine, the parasites acquired further mutations including in the *crt* gene, which conferred higher-level dihydroartemisinin-piperaquine resistance. KEL1/PLA1 subgroups possessing these *crt* mutations were able to spread rapidly across borders in the 2015–18 period. The timing of Thailand's adoption of dihydroartemisinin-piperaquine, driven by concerns about the efficacy of artesunate-mefloquine on the border with Myanmar, was particularly unfortunate as it coincided with the KEL1/PLA1 cross-border expansion. Even in Laos, where artemether-lumefantrine has been the recommended first-line drug since 2005, KEL1/PLA1 parasites have successfully colonised the southernmost province of Champasak, possibly because of cross-border importation of dihydroartemisinin-piperaquine or because of their resistance to the artemisinin component of artemether-lumefantrine.

These findings show an evolutionary process in action. Artemisinin resistance first began as delayed parasite clearance, caused by many mutually exclusive *kelch13* mutations, generally at low frequency and geographically restricted. Over time, a single *kelch13* mutation (C580Y) has become dominant in eastern southeast Asia, in association with several other variants (eg, in *ferredoxin, arps10, mdr2*, and *crt*).^11^, ^12^ Thus, a soft sweep (many different, independently emerging advantageous *kelch13* mutations) became a hard sweep of the *kelch13* C580Y variant as the KEL1/PLA1 co-lineage, resistant to dihydroartemisinin-piperaquine, rose in frequency and swept through Cambodia.^13^ Following that harder sweep, the parasites diversified into separate evolutionary branches with emerging new properties. This perhaps reflects a general tendency for diversification after a hard sweep, as the population explores a vast evolutionary space, acquiring new mutations. The genetic background that has accumulated in eastern southeast Asia appears to underpin the newly emerging alleles in *crt*, as multiple alleles have arisen independently within the past few years that are absent elsewhere. It remains to be seen whether one of these newly emerging alleles will become dominant and drive a new hard sweep, as *kelch13* C580Y did.

The spread of KEL1/PLA1 up to 2013 described by Amato and colleagues^13^ has worsened substantially. By analogy with cancer biology, KEL1/PLA1 can be viewed as an aggressive cell line that has metastasised, invading new territories and acquiring new genetic properties. These patterns emphasise the importance of surveillance in guiding and accelerating malaria elimination. Prolonged use of dihydroartemisinin-piperaquine after resistance first emerged might have created the selective pressure for the evolution of enhanced KEL1/PLA1 subgroups. Given the spread and intensification of resistance, effective translation of genetic surveillance results is crucial to support timely decisions on first-line therapies. Many of the samples in this dataset were obtained by amalgamating data from multiple varied studies, resulting in temporal and geographical heterogeneity that can limit the inferential power. Going forward, there is a need for systematic longitudinal surveillance, and this is now being done by malaria control programmes in Cambodia, Laos, and Vietnam, which contributed many of the recent samples included in this study. These findings highlight the importance of longitudinal genetic surveillance in guiding the elimination of multidrug-resistant *P falciparum* from the Greater Mekong Subregion, and control and elimination efforts elsewhere.
