# Supplementary material for: Evolution and expansion of multidrug-resistant malaria in southeast Asia: a genomic epidemiology study
Source: Lancet Infect Dis. 2019 Sep;19(9):943–51. doi: 10.1016/S1473-3099(19)30392-5 (PMC6715858; doi:10.1016/S1473-3099(19)30392-5)

THE LANCET Infectious Diseases

Supplementary webappendix

This webappendix formed part of the original submission and has been peer reviewed. We post it as supplied by the authors.

Supplement to: Hamilton WL, Amato R, van der Pluijm RW, et al. Evolution and expansion of multidrug-resistant malaria in southeast Asia: a genomic epidemiology study. *Lancet Infect Dis* 2019; published online July 22. http://dx.doi.org/10.1016/S1473-3099(19)30392-5.

Appendix

Evolution and expansion of multidrug resistant malaria in Southeast Asia: a genomic epidemiology study

William L Hamilton, Roberto Amato, Rob W van der Pluijm, Christopher G Jacob, Huynh Hong Quang, Nguyen Thanh Thuy-Nhien, Tran Tinh Hien, Bouasy Hongvanthong, Keobouphaphone Chindavongsa, Mayfong Mayxay, Rekol Huy, Rithea Leang, Cheah Huch, Lek Dysoley, Chanaki Amaratunga, Seila Suon, Rick M Fairhurst, Rupam Tripura, Thomas J Peto, Yok Sovann, Podjanee Jittamala, Borimas Hanboonkunupakarn, Sasithon Pukrittayakamee, Nguyen Hoang Chau, Mallika Imwong, Mehul Dhorda, Ranitha Vongpromek, Xin Hui S Chan, Richard J Maude, Richard D Pearson, T Nguyen, Kirk Rockett, Eleanor Drury, Sónia Gonçalves, Nicholas J White, Nicholas P Day, Dominic P Kwiatkowski, Arjen M Dondorp, Olivo Miotto

Contents

Counts of samples analysed for the different ESEA regions surveyed. 1
Sample numbers included in main analysis, broken down by country and year. 2
Distribution by region of 1,673 samples in the three analysed periods. 3
Haplotypes surrounding the *plasmepsin-II* and *plasmepsin-III* loci for PLA1 samples (A) and non-PLA1 samples (B). 4
Proportion of KEL1/PLA1 parasites per year in each ESEA region. 5
Frequency of parasites with KEL1 and PLA1 haplotypes in the periods 2010-2011 and 2016-2017, corrected for sampling heterogeneity. 6
Frequency of KEL1/PLA1 parasites in the periods 2010-2011 and 2016-2017 for different ESEA regions, corrected for sampling heterogeneity. 7
Low haplotype diversity in KEL1/PLA1 parasites. 8
High degree of similarity among KEL1/PLA1 parasites, regardless of geographical origin. 9
Histogram of pairwise genetic distances for KEL1/PLA1 samples, with lower quartile (used to define related subgroups) marked 10
Haplotype diversity in KEL1/PLA1 subgroups. 11
KEL1/PLA1 family tree, geo-temporal distribution and *crt* alleles. 12
Characteristics of identified KEL1/PLA1 subgroups. 13
Variations in frequency of *crt* mutations between the periods 2010-2011 and 2016-2017, corrected for sampling heterogeneity. 14
Association of *crt* alleles with specific genetic backgrounds. 15
Haplotype diversity in parasites with *crt* Newly Emerging Alleles. 16
Haplotype frequency plots (above) and extended haplotype homozygosity (EHH, below) along the flanking regions of *crt* on chromosome 7 17
Haplotypes across 200KB surrounding the *crt* locus on chromosome 7 18

Counts of samples analysed for the different ESEA regions surveyed.

Region			Sample Count
**Symbol**	**Name**	**Provinces**	
WKH	W Cambodia	Pailin, Pursat, Battambang	518
NKH	N Cambodia	Preah Vihear	126
NEKH	NE Cambodia	Stung Treng, Ratanakiri	280
NETH	NE Thailand	Sisaket	41
LA	Lao PDR	Savannakhet, Salavan, Sekong, Attapeu, Champasak	250
VN	Vietnam	Binh Phuoc, Dak Lak, Dak Nong, Gia Lai, Quang Tri, Ninh Thuan, Khanh Hoa	458
		**Total**	**1673**

Sample numbers included in main analysis, broken down by country and year.
The regions and their symbols are detailed on pages 1 and 5, and displayed on a map on page 3.

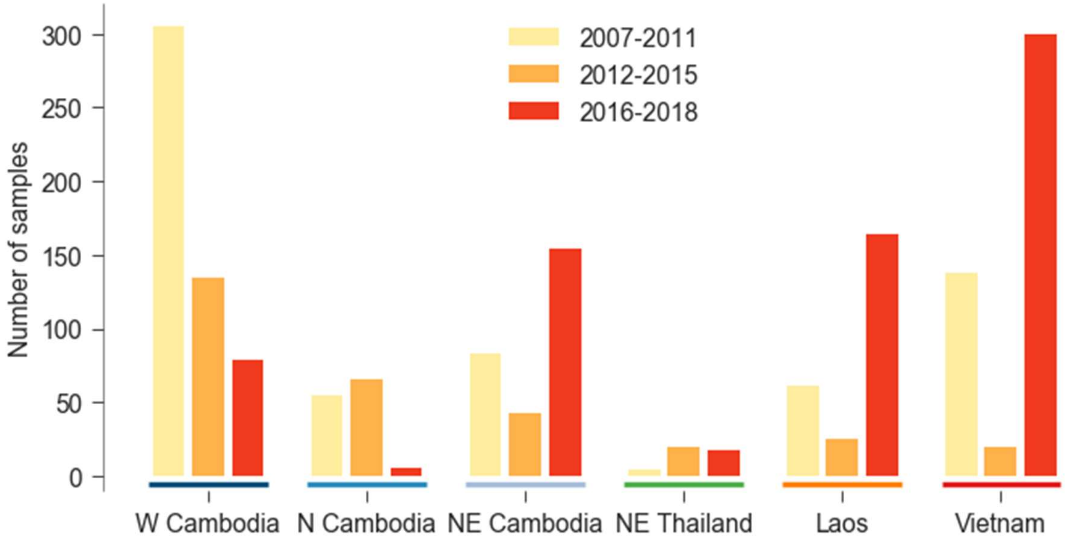

Distribution by region of 1,673 samples in the three analysed periods.
Markers are coloured by region, and their size reflects the number of samples (shown in the labels below each marker).

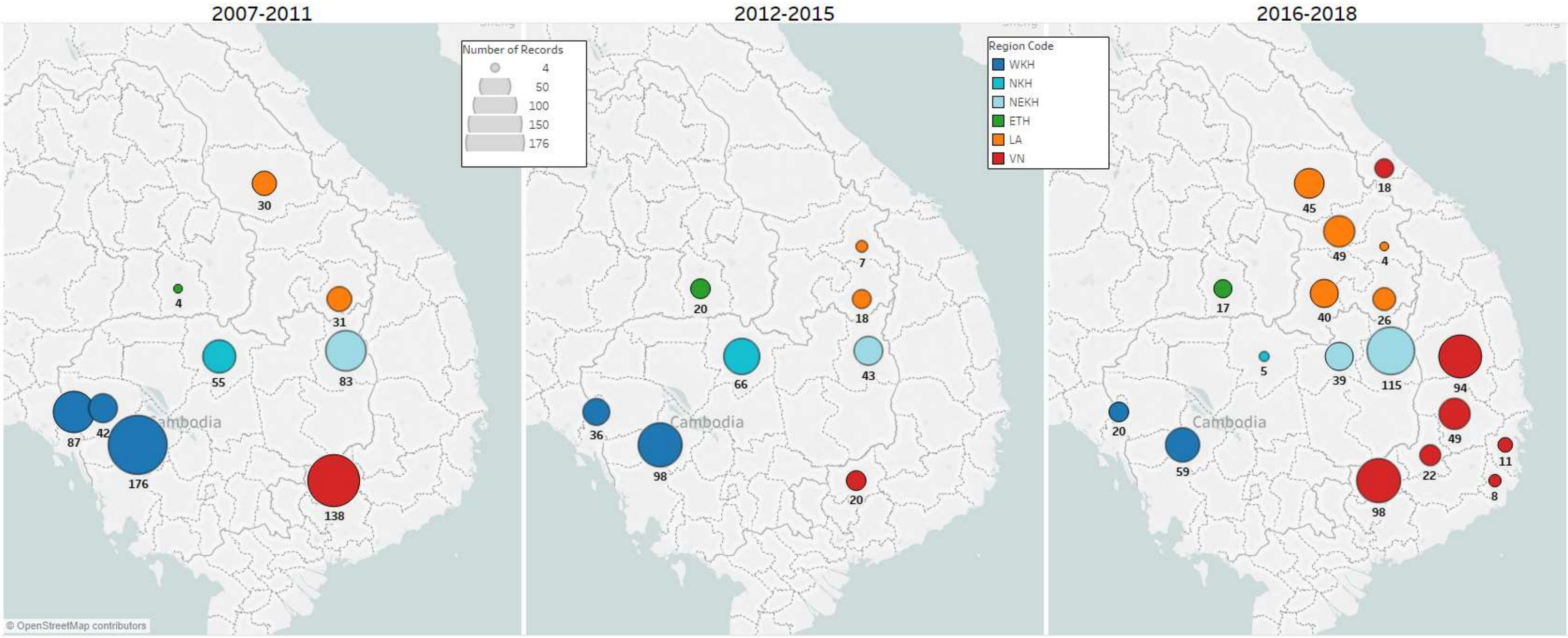

Haplotypes surrounding the *plasmepsin-II* and *plasmepsin-III* loci for PLA1 samples (A) and non-PLA1 samples (B).

Each row represents a sample, and each column represents a single SNP variant. Cells are coloured white for the reference allele (same as 3D7 reference sequence) and black for non-reference allele. The red lines enclose SNPs in the two *plasmepsin* genes. The presence of a single shared haplotype surrounding these loci is consistent with a single epidemiological origin of the genetic background on which the amplification arose.

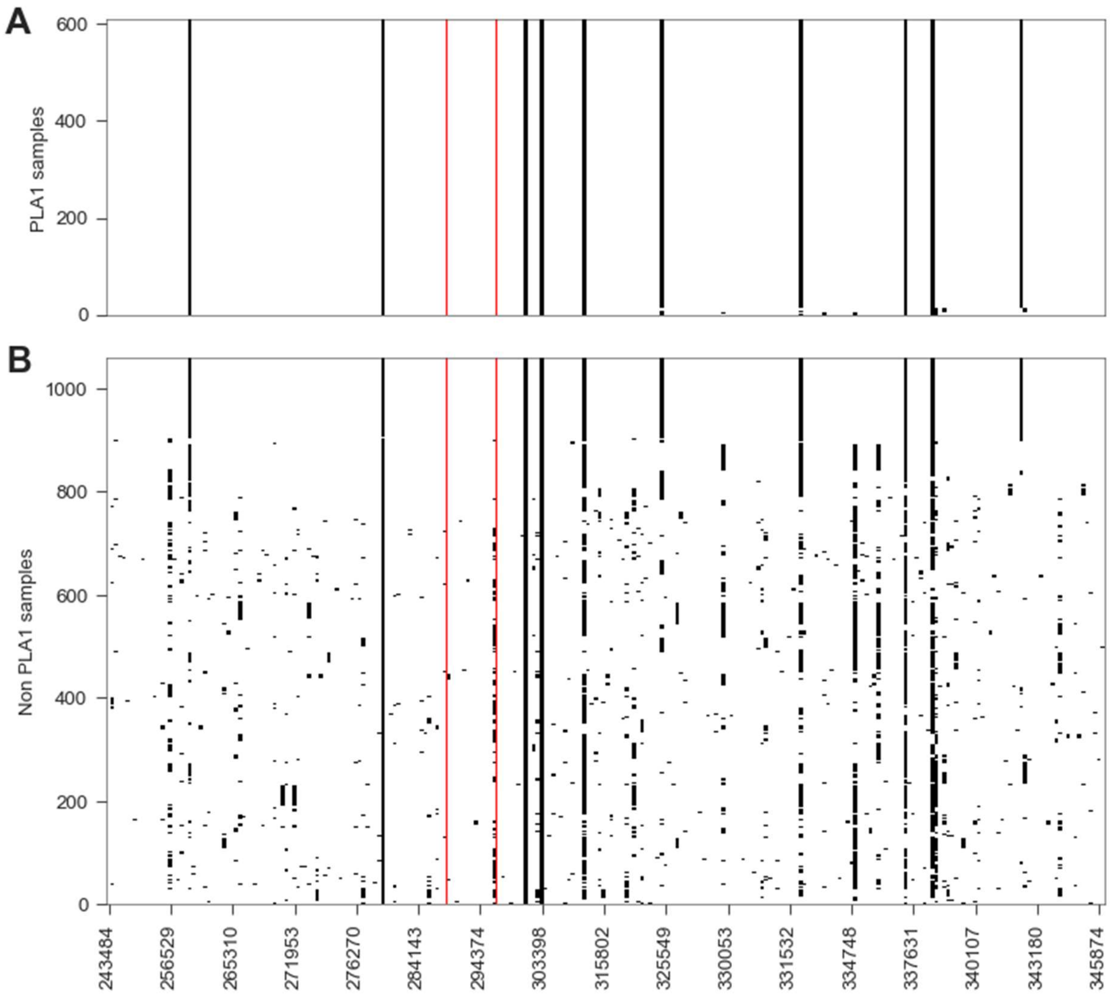

Proportion of KEL1/PLA1 parasites per year in each ESEA region.

Absolute numbers of samples are shown in parenthesis. Dashes indicate that no samples were available in our dataset for that region/year combination.

Year	W Cambodia	N Cambodia	NE Cambodia	NE Thailand	Lao PDR	Vietnam
**2007**	0% (0/6)	-	100% (1/1)	-	-	-
**2008**	15% (11/74)	-	-	-	-	-
**2009**	41% (9/22)	-	-	-	-	0% (0/9)
**2010**	23% (19/82)	-	0% (0/33)	-	0% (0/30)	0% (0/54)
**2011**	39% (47/121)	0% (0/55)	0% (0/49)	0% (0/4)	0% (0/31)	0% (0/75)
**2012**	58% (28/48)	8% (3/38)	0% (0/18)	0% (0/11)	0% (0/18)	0% (0/20)
**2013**	56% (20/36)	27% (6/22)	14% (1/7)	0% (0/2)	-	-
**2014**	72% (18/25)	80% (4/5)	6% (1/18)	-	-	-
**2015**	84% (21/25)	100% (1/1)	-	100% (7/7)	0% (0/7)	-
**2016**	78% (28/36)	100% (5/5)	47% (39/83)	100% (1/1)	0% (0/3)	69% (34/49)
**2017**	68% (28/41)	-	60% (41/68)	88% (14/16)	20% (32/161)	51% (128/251)
**2018**	100% (2/2)	-	67% (2/3)	-	-	-

Frequency of parasites with KEL1 and PLA1 haplotypes in the periods 2010-2011 and 2016-2017, corrected for sampling heterogeneity.

For each time period, we show median haplotype frequency (with interquartile range, IQR) estimated from 50 randomly selected samples from each region (except northeast Thailand and north Cambodia, for which sample size was insufficient), repeated for 100 iteration.

Haplotype status	2010-2011	2016-2017
**Neither KEL1 nor PLA1**	80.62%<br>(79.46% - 81.79%)	29.02%<br>(26.91% - 30.97%)
**PLA1 only**	1.03%<br>(0.52% - 1.55%)	4.18%<br>(3.63% - 4.67%)
**KEL1 only**	9.57%<br>(8.29% - 10.42%)	14.95%<br>(13.92% - 16.21%)
**KEL1/PLA1**	8.38%<br>(7.73% - 9.38%)	51.66%<br>(50.00% - 53.42%)

Frequency of KEL1/PLA1 parasites in the periods 2010-2011 and 2016-2017 for different ESEA regions, corrected for sampling heterogeneity.

For each time period, we show median haplotype frequency (with interquartile range, IQR) estimated from 50 randomly selected samples from each region (except northeast Thailand and north Cambodia, for which sample size was insufficient), repeated for 100 iteration.

Region	2010-2011	2016-2017
**W Cambodia**	34.00% (31.25% - 37.62%)	74.00% (71.43% - 75.63%)
**NE Cambodia**	0.00% (0.00% - 0.00%)	56.25% (52.15% - 61.34%)
**Laos**	0.00% (0.00% - 0.00%)	20.00% (18.00% - 22.45%)
**Vietnam**	0.00% (0.00% - 0.00%)	56.83% (52.84% - 60.96%)

Low haplotype diversity in KEL1/PLA1 parasites.

Boxplots show the distribution of haplotype diversity measures in rolling 100-SNP windows with 50-SNP overlaps along the whole genome for populations of KEL1/PLA1 (red, left) and non-KEL1/PLA1 (blue, right) parasites in the different regions studied. Haplotype diversity is the proportion of SNP windows that have at least 1 SNP difference between samples, where zero means all SNP-windows are identical and 1 means all have at least 1 SNP difference. Thick lines represent median values, boxes show the interquartile range, whiskers represent extremes of the distribution discounting outliers. Pairwise comparisons between the KEL1/PLA1 and non-KEL1/PLA1 groups for each region were all statistically significant with Bonferroni correction for multiple comparisons ($P<10^{-16}$, Mann-Whitney *U* test).

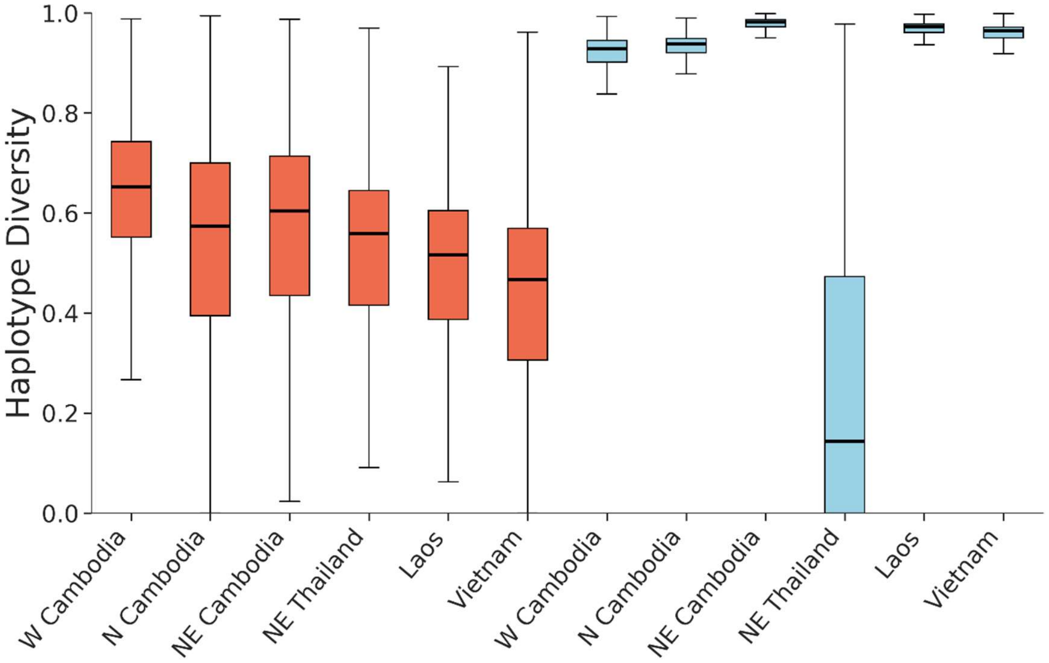

High degree of similarity among KEL1/PLA1 parasites, regardless of geographical origin.

Principal Component Analysis (PCoA) based on genetic distance, coloured by country (A) and KEL1/PLA1 status (B). Genetic distances were calculated with correction for linkage disequilibrium and minor allele frequency cutoff of 1%.

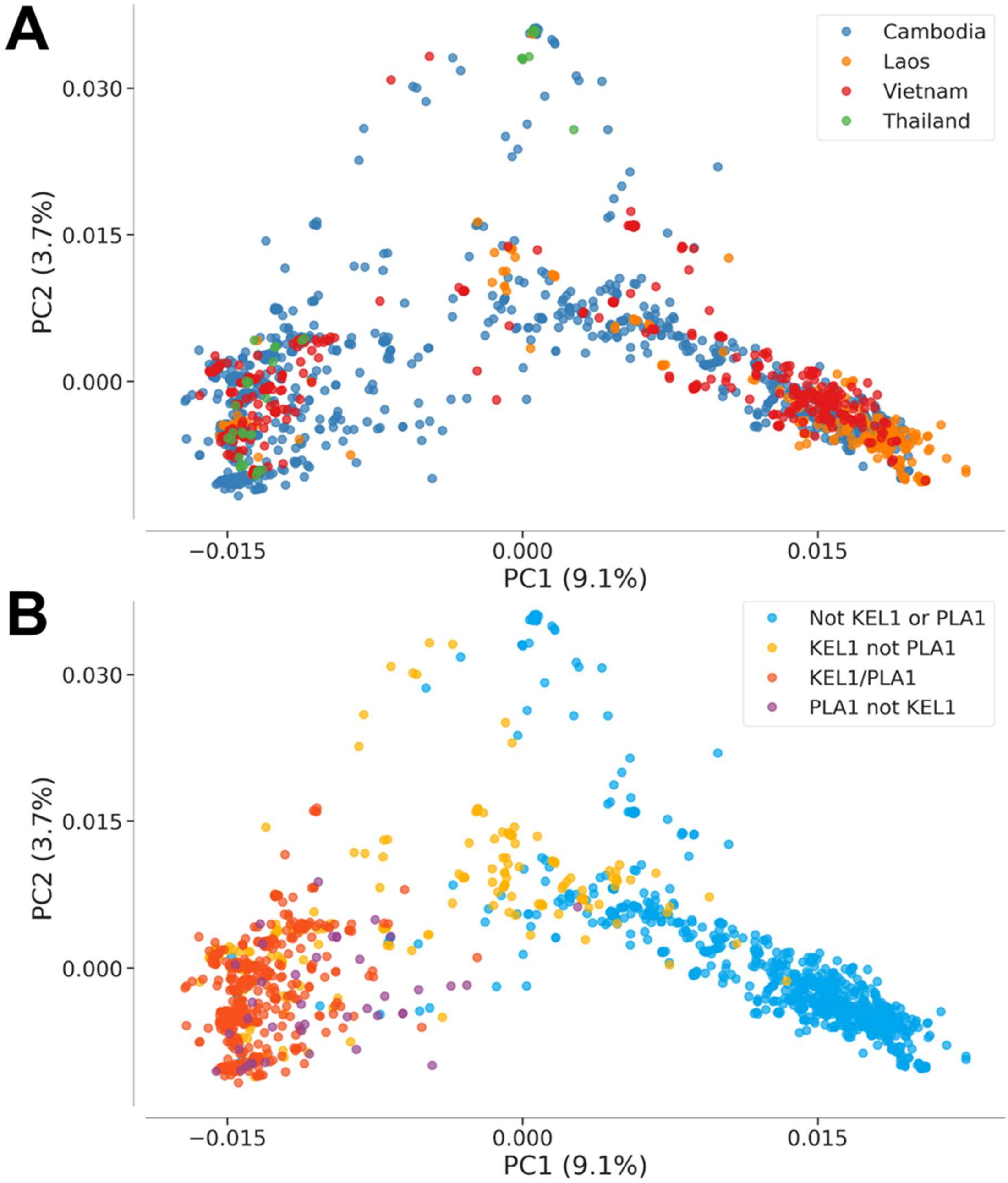

Histogram of pairwise genetic distances for KEL1/PLA1 samples, with lower quartile (used to define related subgroups) marked.

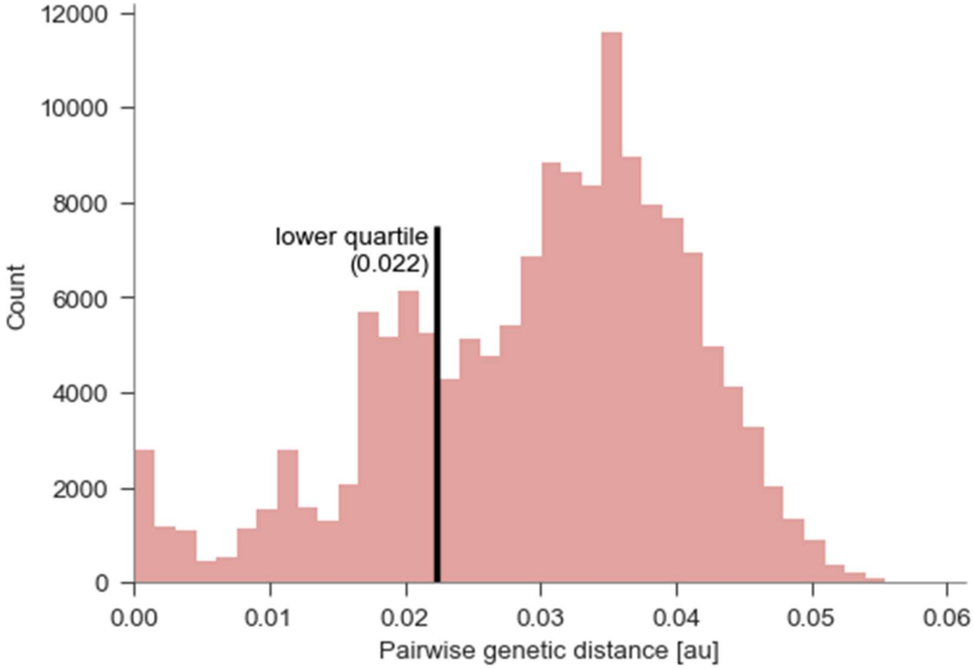

Haplotype diversity in KEL1/PLA1 subgroups.

Boxplots show the distribution of haplotype diversity in different parasite groups, measured in rolling 100-SNP windows with 50-SNP overlaps along the whole genome . Haplotype diversity is the proportion of SNP windows that have at least 1 SNP difference between samples, where zero means all SNP-windows are identical and 1 means all have at least 1 SNP difference. Thick lines represent median values, boxes show the interquartile range, whiskers represent extremes of the distribution discounting outliers.

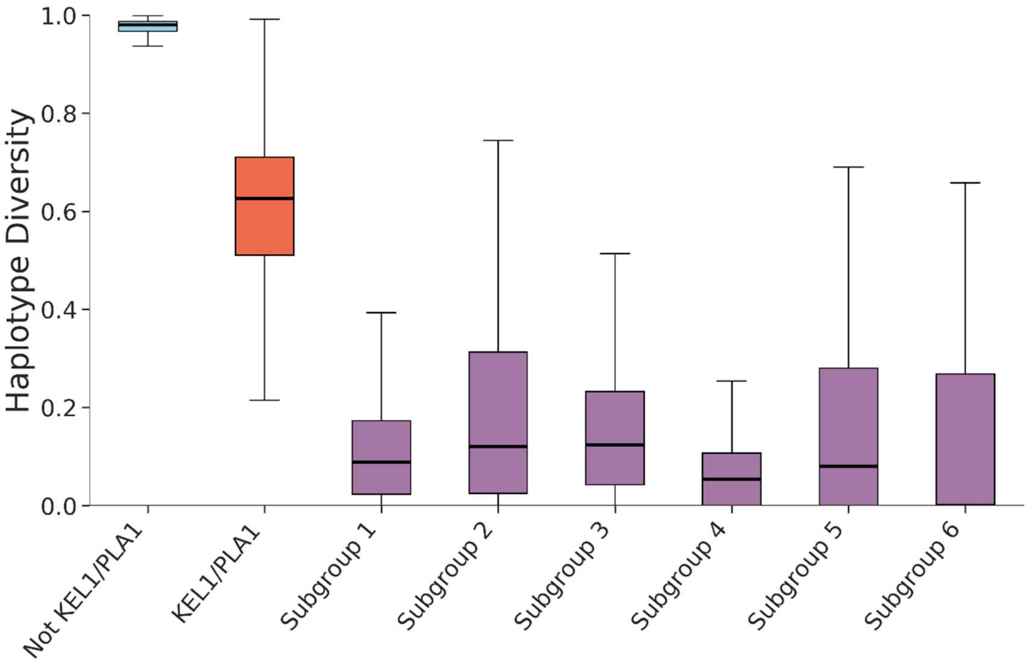

KEL1/PLA1 family tree, geo-temporal distribution and *crt* alleles.

(A) Dendrogram of genetic distances within all 551 analysed KEL1/PLA1 samples across ESEA, identical to that shown in Figure 3. The six largest subgroups are highlighted and labelled. Colour bars indicate sampling region (B), year (C) and the presence of any Newly Emerging Alleles in *crt*, as defined in main text (D). Pairwise genetic distance is expressed in an arbitrary unit which is a function of the number of genetic differences observed among variant SNPs in this dataset between pairs of samples, after correcting for linkage disequilibrium and heterozygous genotypes.

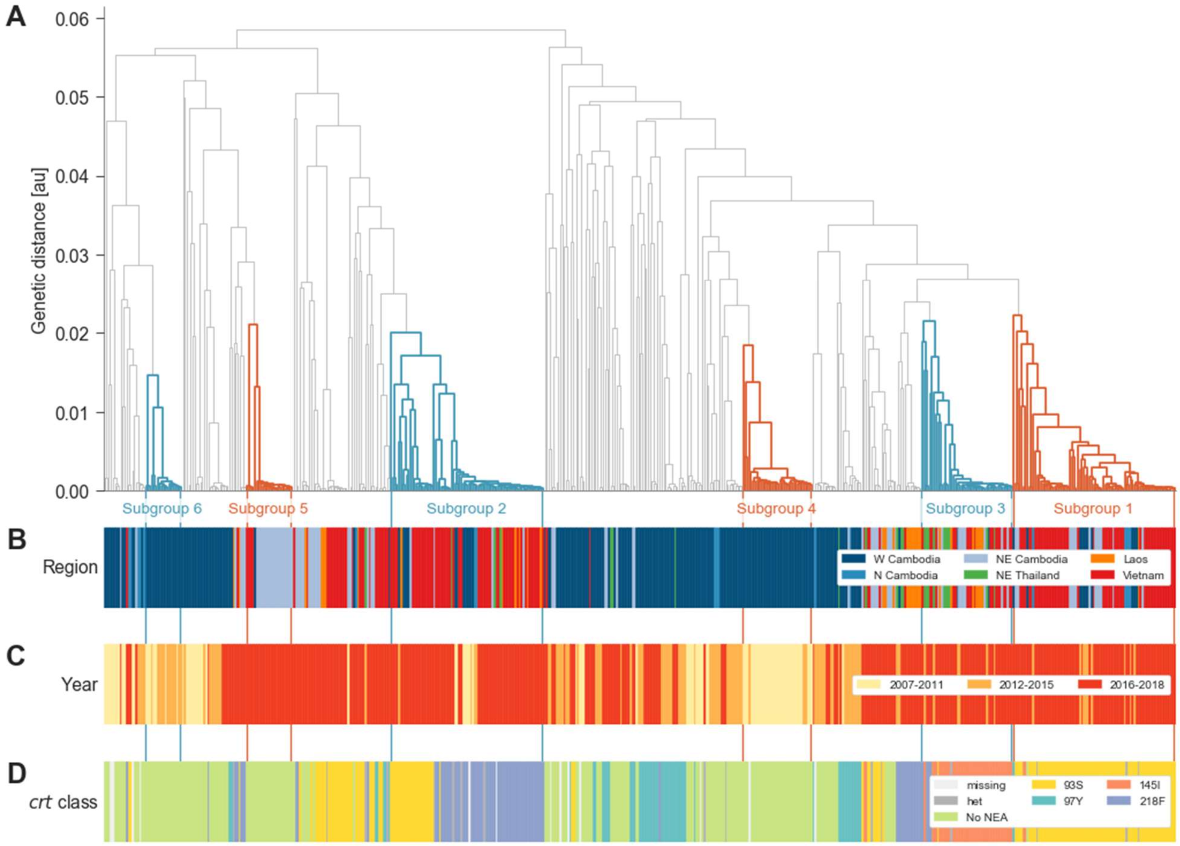

Characteristics of identified KEL1/PLA1 subgroups.

For each of the subgroups containing at least 5 samples, we show its distribution by region (left), year of sampling (middle) and *crt* haplotype (right). Parasites classified as "*undetermined*" in the latter section had insufficient coverage of the *crt* gene to determine their haplotype reliably or had ambiguous (het) calls.

Subgroup	Region						Collection year			*crt* haplotype					
	WKH	NKH	NEKH	NETH	LA	VN	2007-2011	2012-2015	2016-2018	T93S	H97Y	F145I	I218F	No NEA	Undetermined
**1**	11	5	13	0	1	54	0	9	75	77	1	2	0	1	3
**2**	13	2	4	3	6	51	7	4	68	23	0	0	50	0	6
**3**	1	1	9	10	7	19	0	6	41	1	0	41	4	0	1
**4**	36	0	0	0	0	0	33	3	0	0	0	0	0	35	1
**5**	1	0	19	0	0	4	0	0	24	0	0	0	0	24	0
**6**	19	0	0	0	0	0	7	12	0	0	0	0	0	19	0
**7**	0	0	9	1	3	4	0	0	17	7	0	2	0	6	2
**8**	0	0	0	0	3	11	0	0	14	13	0	0	0	0	1
**9**	1	0	0	3	8	1	0	2	11	0	0	0	13	0	0
**10**	11	1	0	0	0	0	0	8	4	0	1	0	0	10	1
**11**	9	1	1	1	0	0	1	10	1	0	12	0	0	0	0
**12**	12	0	0	0	0	0	1	6	5	0	0	0	1	11	0
**13**	11	0	0	0	0	0	0	8	3	0	0	0	0	10	1
**14**	7	0	1	2	0	0	0	1	9	0	0	0	0	10	0
**15**	3	5	1	0	0	1	2	4	4	2	0	0	1	5	2
**16**	8	0	0	1	0	0	0	6	3	0	9	0	0	0	0
**17**	1	0	0	0	0	7	0	0	8	2	5	0	0	0	1
**18**	4	3	0	0	0	0	2	5	0	0	0	0	0	7	0
**19**	0	0	5	0	0	0	0	0	5	4	0	0	0	1	0
**20**	5	0	0	0	0	0	4	1	0	0	1	0	0	4	0

Variations in frequency of *crt* mutations between the periods 2010-2011 and 2016-2017, corrected for sampling heterogeneity.

For each time period, we show median mutation frequency (with interquartile range, IQR) estimated from 50 randomly selected samples from each region (except northeast Thailand and north Cambodia, for which sample size was insufficient), repeated for 100 iteration. Mutations labelled as "NEA" are those absent in the first period (frequency ≤ 1%) and increased by >5% in the second period. Mutations below 1% frequency in at least one period were disregarded.

crt variant	Frequency in 2010-2011	Frequency in 2016-2017	Frequency Change	
**M74I**	91.96% (91.00% - 92.50%)	97.24% (96.50% - 97.99%)	5.49% (4.48% - 6.27%)	
**N75D**	75.91% (73.85% - 77.61%)	79.67% (75.49% - 84.80%)	4.40% (-0.93% - 9.36%)	
**N75E**	89.06% (87.92% - 90.01%)	96.95% (96.15% - 97.73%)	7.86% (6.67% - 9.02%)	
**K76T**	91.96% (91.00% - 92.50%)	97.24% (96.50% - 97.99%)	5.49% (4.48% - 6.27%)	
**T93S**	**0.00% (0.00% - 0.00%)**	**19.75% (18.00% - 21.39%)**	**19.75% (18.00% - 21.39%)**	**NEA**
**H97Y**	**1.00% (0.50% - 1.50%)**	**11.20% (10.41% - 11.87%)**	**10.25% (9.16% - 11.23%)**	**NEA**
**H97L**	0.00% (0.00% - 0.50%)	1.71% (1.13% - 2.27%)	1.70% (1.12% - 2.27%)	
**A144F**	25.50% (24.50% - 26.80%)	10.83% (9.60% - 12.31%)	-15.02% (-16.34% - -13.29%)	
**F145I**	**0.00% (0.00% - 0.00%)**	**5.54% (4.54% - 6.57%)**	**5.54% (4.54% - 6.57%)**	**NEA**
**L148I**	25.51% (24.37% - 26.78%)	10.86% (9.71% - 12.31%)	-14.89% (-16.27% - -13.13%)	
**I194T**	27.23% (25.87% - 28.27%)	15.31% (13.73% - 17.64%)	-11.80% (-13.73% - -9.15%)	
**I218F**	**0.77% (0.51% - 1.04%)**	**11.14% (9.99% - 12.20%)**	**10.28% (9.15% - 11.35%)**	**NEA**
**A220S**	91.69% (90.76% - 92.31%)	97.34% (96.68% - 97.98%)	5.74% (4.72% - 6.48%)	
**Q271E**	91.96% (91.00% - 92.50%)	97.27% (96.57% - 97.93%)	5.44% (4.38% - 6.22%)	
**H273N**	1.01% (0.50% - 1.51%)	0.51% (0.00% - 1.02%)	-0.50% (-1.00% - 0.00%)	
**N326S**	38.61% (37.41% - 39.45%)	76.49% (74.35% - 77.96%)	38.03% (35.96% - 40.01%)	
**T333S**	25.38% (24.37% - 26.67%)	11.08% (9.88% - 12.53%)	-14.75% (-15.89% - -12.82%)	
**M343I**	0.00% (0.00% - 0.00%)	2.26% (2.00% - 2.75%)	2.26% (2.00% - 2.75%)	
**G353V**	1.01% (0.50% - 1.50%)	4.52% (4.50% - 5.03%)	3.99% (3.02% - 4.50%)	
**I356T**	39.45% (38.39% - 40.42%)	77.27% (75.00% - 78.50%)	37.66% (35.85% - 39.53%)	
**R371I**	66.50% (65.25% - 68.25%)	86.36% (84.91% - 87.94%)	19.46% (17.83% - 21.94%)	

Association of *crt* alleles with specific genetic backgrounds.

Each column represents a circulating mutation in the *crt* gene, and each row represents a genetic background, as mentioned in the main text. Each cell is coloured according to the frequency of the mutation (column) in parasites that carry the specified haplotype (row). NEAs arise on a genetic background comprising the chloroquine resistant CVIET haplotype, the additional mutations at positions 326 and 356, and the KEL1/PLA1 haplotype. Three other mutually exclusive crt alleles found at lower frequencies than the NEAs (H97L, M343I and G353V) were also associated with the same genetic background as the NEAs, though H97L only occurred in non-PLA1 parasites.

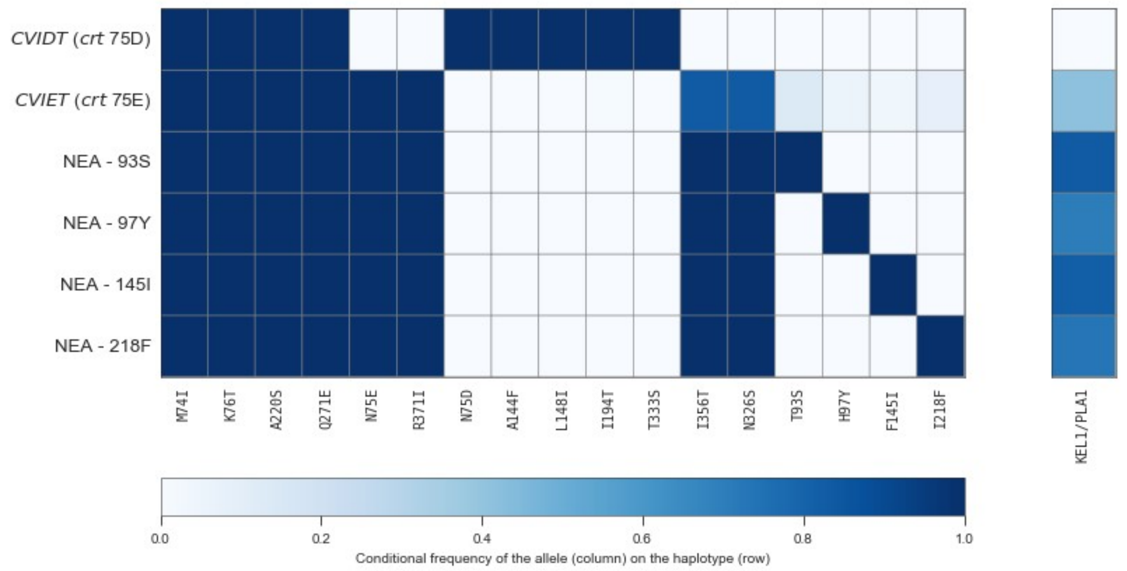

Haplotype diversity in parasites with *crt* Newly Emerging Alleles.

Boxplots show the distribution of haplotype diversity in parasites possessing different crt Newly Emerging Alleles, and parasites without NEAs, measured in rolling 100-SNP windows with 50-SNP overlaps along the whole genome (A) and just chromosome 7 (B), where *crt* is situated. Haplotype diversity is the proportion of SNP windows that have at least 1 SNP difference between samples, where zero means all SNP-windows are identical and 1 means all have at least 1 SNP difference. Thick lines represent median values, boxes show the interquartile range, whiskers represent extremes of the distribution discounting outliers. Pairwise comparisons between "no NEA" and each NEA groups were all statistically significant with Bonferroni correction for multiple comparisons ($P<10^{-16}$, Mann-Whitney *U* test).

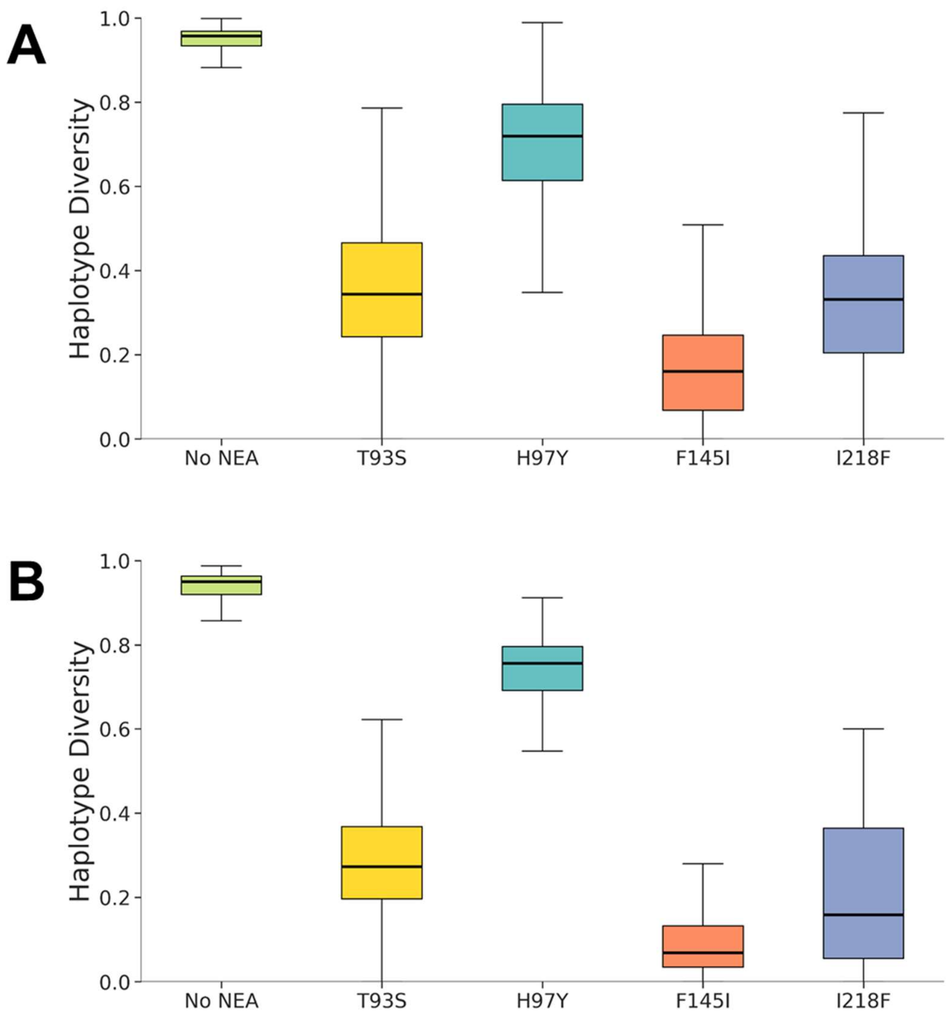

Haplotype frequency plots (above) and extended haplotype homozygosity (EHH, below) along the flanking regions of *crt* on chromosome 7

Samples are from each of the following allele groups: no NEA (A), T93S (B), H97Y (C), F145I (D) and I218F (E). The colours have no specific meaning and simply depict different haplotypes.

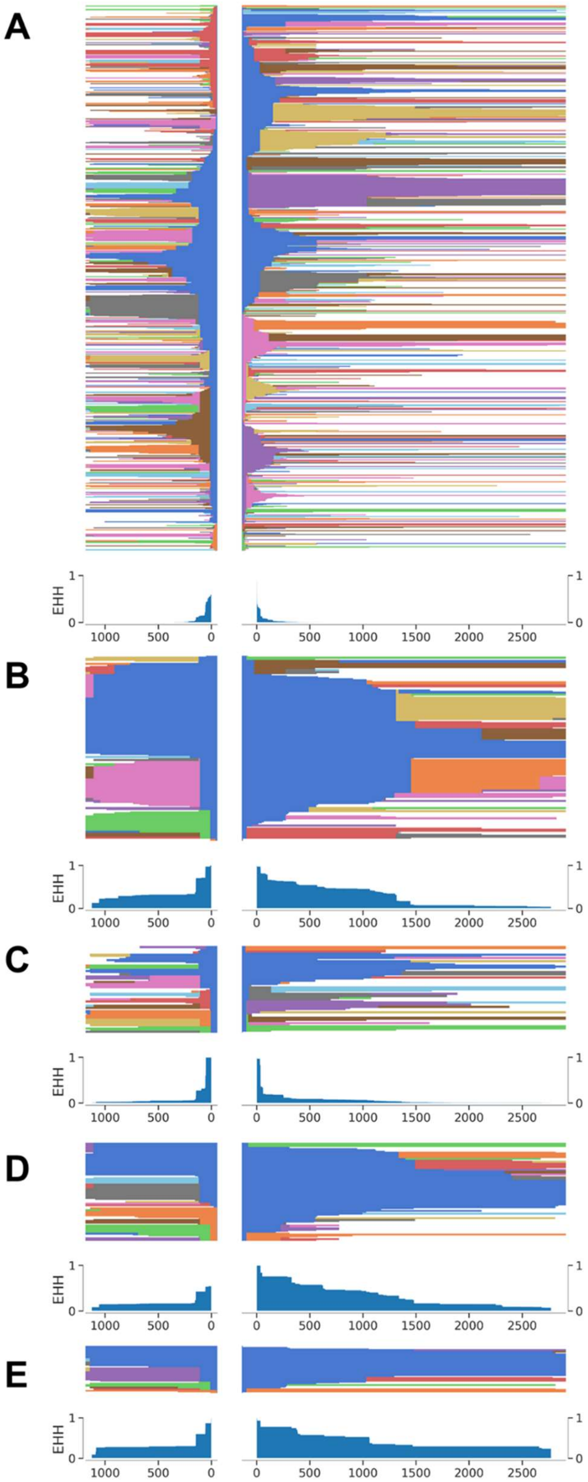

Haplotypes across 200KB surrounding the *crt* locus on chromosome 7

Samples are shown for each of the following allele groups: no NEA (A), T93S (B), H97Y (C), F145I (D) and I218F (E). All mutant samples are included for the NEAs (panels B-E), and a randomised subset of 100 no-NEA samples is shown in Panel A. Black represents non-reference alleles and white is reference. SNPs bordering the *crt* gene are marked in red.

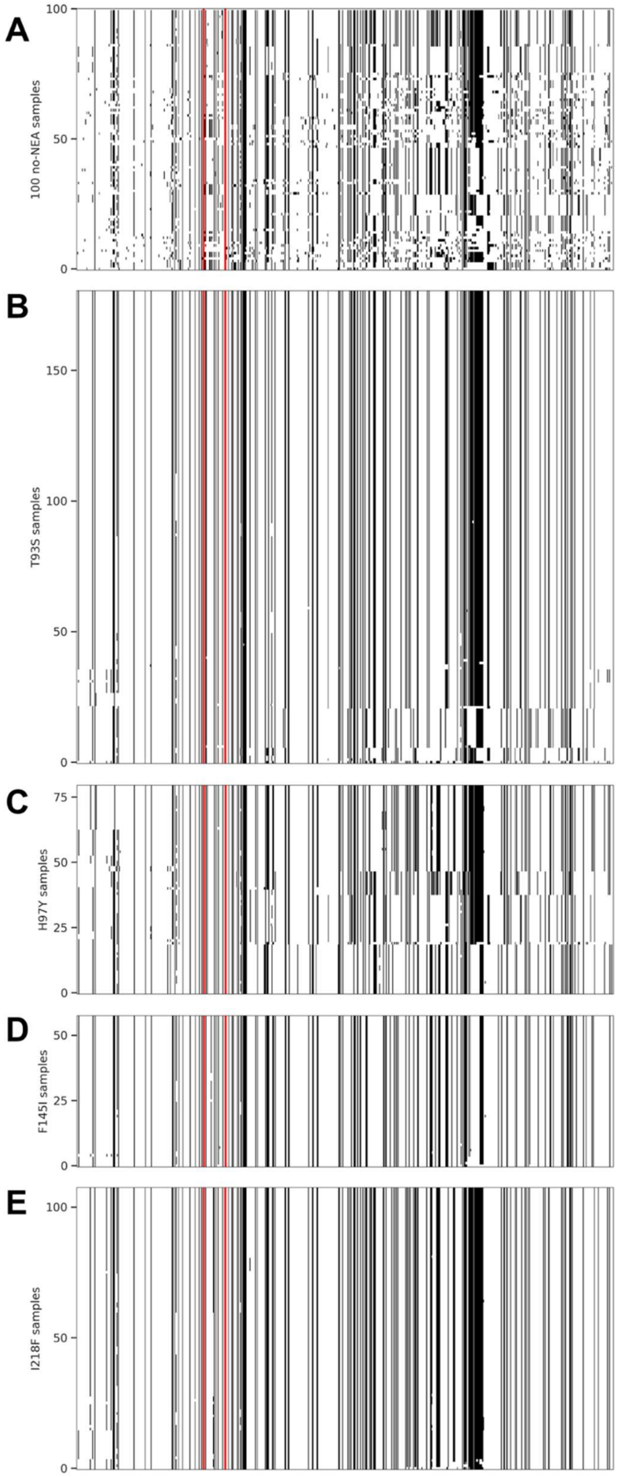

Supplement: Supplementary appendix [file mmc1.pdf]
